# Design and Control of Bio-Inspired Joints for Legged Robots Driven by Shape Memory Alloy Wires

**DOI:** 10.3390/biomimetics10060378

**Published:** 2025-06-06

**Authors:** Xiaojie Niu, Xiang Yao, Erbao Dong

**Affiliations:** 1 Institute of Advanced Technology, University of Science and Technology of China, Hefei 230026, China; mr12@mail.ustc.edu.cn; 2 School of Engineering Science, University of Science and Technology of China, Hefei 230026, China; yaoxiangyx@mail.ustc.edu.cn

**Keywords:** shape memory alloy, bio-inspired joint, bio-inspired leg, motion control

## Abstract

Bio-inspired joints play a pivotal role in legged robots, directly determining their motion capabilities and overall system performance. While shape memory alloy (SMA) actuators present superior power density and silent operation compared to conventional electromechanical drives, their inherent nonlinear hysteresis and restricted strain capacity (typically less than 5%) limit actuation range and control precision. This study proposes a bio-inspired joint integrating an antagonistic actuator configuration and differential dual-diameter pulley collaboration, achieving amplified joint stroke (±60°) and bidirectional active controllability. Leveraging a comprehensive experimental platform, precise reference input tracking is realized through adaptive fuzzy control. Furthermore, an SMA-driven bio-inspired leg is developed based on this joint, along with a motion retargeting framework to map human motions onto the robotic leg. Human gait tracking experiments conducted on the leg platform validate its motion performance and explore practical applications of SMA in robotics.

## 1. Introduction

Owing to the superior properties of shape memory alloy (SMA), SMA-actuated drivers demonstrate significant advantages over conventional actuation methods such as electromagnetic, hydraulic, and pneumatic systems in terms of power-to-weight ratio, output force, and silent operation, effectively addressing the stringent spatial and weight constraints in robotic applications [1,2]. Consequently, researchers worldwide have conducted extensive work on the design and control of SMA actuators.

Regarding SMA actuator design, various rotational and linear actuators have been developed through innovative structural configurations to achieve diverse motion patterns in the past [3,4,5,6,7,8,9,10,11,12,13,14]. These studies are distributed over a long period of time, but both new and old works have inspired our study in terms of structural design and driver configuration. Simultaneously, researchers have successfully implemented SMA actuators in various robotic systems [9,10,15,16,17,18], expanding their application scope and demonstrating preliminary practical implementations of SMA-driven solutions in robotic platforms.

Regarding the control of SMA actuators, while numerous successful implementations have been demonstrated [1,19,20,21,22,23,24], the inherent pronounced nonlinearities [25] and limited contraction rates of SMAs necessitate a holistic design approach integrating structural, control, and hardware considerations. This multidimensional optimization makes the investigation of SMA applications in robotics both scientifically significant and practically valuable.

This study focuses on the design of SMA-driven modules, the innovation of SMA-based joint structures, the integration of SMA-actuated bionic leg systems, and the deployment of control algorithms, establishing a comprehensive technical chain from actuation units to application validation. The primary innovations lie in the novel SMA joint configuration and bionic leg system integration. Specifically, a multi-wire parallel SMA drive module is designed, leveraging the intrinsic advantages of SMAs under the principle of reconfigurability. Building on this module, a bionic joint is developed, integrating the antagonistic actuator configuration and differential dual-diameter fixed pulleys to achieve a large motion range and bidirectional active control. Furthermore, an adaptive fuzzy control algorithm is deployed in the designed joint, with extensive experiments demonstrating its superior performance. Finally, a bionic leg system and a motion retargeting method are constructed based on the joint, experimentally validating its potential for human gait tracking. This work provides a promising actuator solution for robotics, bridging fundamental design with practical implementation.

## 2. Design of Multi-Wire Parallel SMA Drive Module

As the power source of the entire bionic joint system, the detailed design and analysis of the drive module are of primary importance. Inspired by bionics, electromechanical structures have permeated various aspects of human life. Similarly, the human body, a system capable of adapting to diverse natural or artificial environments, provides principles deconstructed from skeletal muscle configuration and joint structures, which can effectively guide the design of SMA-driven bionic joint systems. The multi-wire parallel SMA drive module, inspired by human skeletal muscles, is a modular actuation system composed of SMA wires.

**Figure 1:**
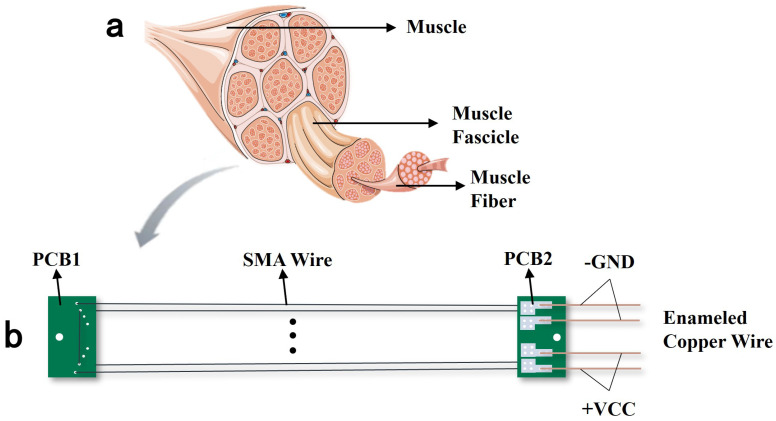
Bio-inspired SMA driving module: (**a**) Schematic diagram of skeletal muscle. (**b**) Multi-wire parallel SMA driving module. Image a is modified from Servier Medical ART, licensed under a Creative Commons Attribution 4.0 generic license (SMART-Servier Medical ART. https://smart.servier.com/, accessed on 17 April 2025).

As illustrated in Figure 1a, skeletal muscles consist of muscle fibers, each serving as the fundamental functional unit. These muscles generate force and control motion through contraction, following a mechanism: a large number of muscle fibers are actuated by neural signals, collectively producing macroscopic changes in joint position, output force, and stiffness at the limb level.

Similarly, SMA wires, as intelligent materials, undergo phase transformation upon thermal excitation driven by control signals, generating stress and strain. In contrast to conventional SMA actuators typically driven by single or a few SMA wires, this study proposes a multi-wire parallel SMA drive module. This module employs parallel configurations of multiple SMA wires to achieve reconfigurable and modular design. Here, reconfigurability refers to the flexible adjustment of the quantity of SMA wire and specifications based on the application requirements to adapt to diverse working environments, while modularity ensures that the drive module operates as an independent unit, compatible with other SMA modules or hybrid actuation systems, thereby enhancing scalability and adaptability. A schematic of the module is shown in Figure 1b.

The module consists of two PCBs, four SMA wires of identical thickness, and four enameled copper wires. Four 0.25 mm diameter thick wires are symmetrically arranged through the outer holes. Detailed dimensional parameters are listed in Table 1. PCB1 and PCB2 are each equipped with 2 mm diameter through-holes for connecting force lines. Additionally, PCB1 features eight symmetrical 0.3 mm holes, through which the SMA wires pass. Notably, the four wires are symmetrically distributed along the module’s axis and across both sides of PCB1, ensuring symmetric force distribution during SMA contraction to prevent flipping or torsion. PCB2 incorporates symmetrically arranged solder pads, which serve to secure the SMA wires and act as voltage input interfaces. Redundant holes and pads on PCB1 and PCB2 allow for the integration of additional SMA wires, enabling reconfigurability to adapt to diverse environments and operational conditions.

A single SMA drive module mimics the actuation characteristics of skeletal muscles, specifically, the rapid response and high energy density through contraction and relaxation. When voltage is applied, Joule heating generated by the inherent resistance of the SMA wire triggers an austenitic phase transformation, resulting in a macroscopic contraction that produces significant stress and strain, analogous to muscle contraction. As temperature decreases, the SMA undergoes a martensitic phase transformation, returning to its original length, corresponding to muscle relaxation. This intrinsic behavior enables SMA drive modules to actuate joint rotation.

## 3. SMA-Driven Bio-Inspired Joint

The SMA-driven bionic joint is a specialized rotary actuator, where the thermally induced contraction of SMA wires drives pulley rotation to generate angular displacement and torque output. However, the inherent limitations of SMAs, unidirectional contraction capability and restricted contraction ratio, pose significant challenges for actuator design. To address these, we introduce a synergistic interaction between an antagonistic actuator configuration and a differential dual-diameter fixed pulley mechanism, enabling large-range motion and bidirectional active control.

### 3.1. Antagonistic Drive Module Configuration

Common SMA rotary actuators primarily employ biased or antagonistic configurations [5]. In a biased configuration, SMA wires are arranged on one side of the joint pulley, while an elastic element (e.g., a spring) is placed on the opposite side. The spring releases elastic potential energy to reset the pulley, simplifying the drive design and avoiding energy consumption. However, this approach allows only unidirectional active control. In contrast, the antagonistic configuration positions SMA wires on both sides of the joint pulley. Through coordinated actuation of the opposing SMA wires, bidirectional active control of the joint is achieved.

### 3.2. Differential Dual-Diameter Fixed Pulley

**Figure 2:**
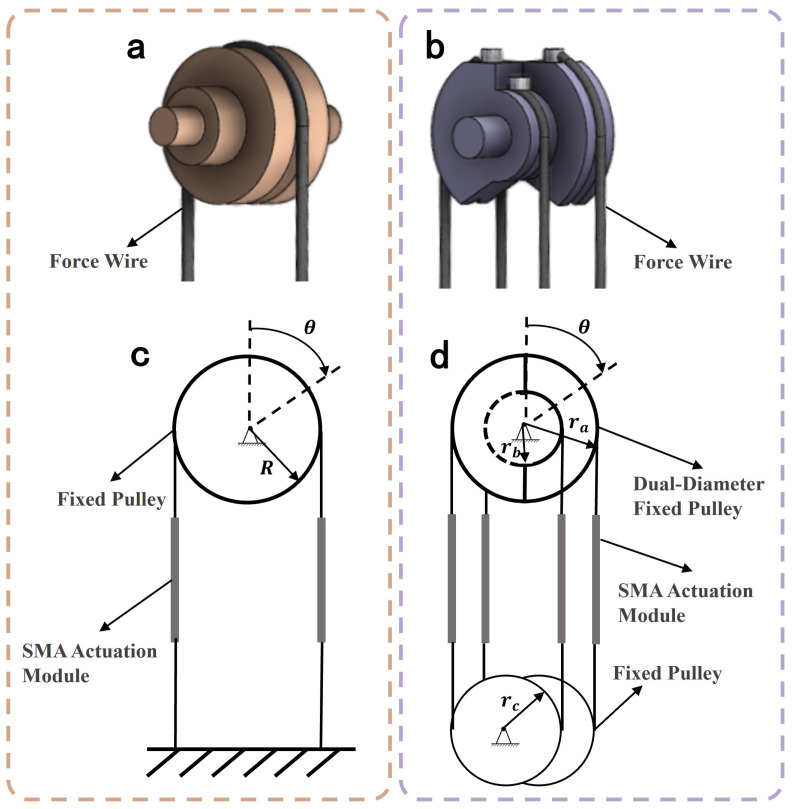
Two design configurations of joint-pulley systems: (**a**) Conventional joint structure diagram. (**b**) Differential joint structure diagram. (**c**) Schematic of conventional joint. (**d**) Schematic of differential joint.

On the other hand, as shown in Figure 2a,c, traditional SMA rotary actuators rely on wire contraction to drive joint pulley rotation. The relationship between the maximum joint rotation angle θ and the maximum wire contraction Δl can be expressed as

(1) $$\theta =\frac{\Delta l}{R}$$

where *R* is the radius of the joint pulley. From this relationship, it can be derived that increasing the motion range of joint requires either lengthening the SMA wires or reducing the pulley radius. However, in spatially constrained systems, the length of the wire cannot be arbitrarily extended. Moreover, decreasing the pulley radius compromises the load-bearing capacity of the joint.

To overcome the performance limitations of traditional single-diameter pulley structures, this section proposes a differential joint pulley integrated with a fixed pulley assembly as illustrated in Figure 2b,d. This structural innovation aims to significantly increase the stroke of the joint. The differential pulley adopts a dual-diameter configuration, combining larger and smaller diameters. In the actual assembly, series-connected SMA drive modules are symmetrically arranged on both axial sides of the pulley, forming an antagonistic configuration. A notched structure is incorporated at the pulley’s base to eliminate mechanical interference during force transmission, ensuring continuous motion during large-angle rotation.

When series-connected SMA modules are thermally activated and contract, the radius discrepancy between the side of the dual-diameter pulley generates a torque differential, driving joint rotation. The kinematic relationship can be expressed as

(2) $$\theta =\frac{\Delta l}{r_{a}-r_{b}}$$

where $r_{a}$ and $r_{b}$ represent the radii of the large-diameter groove and the small-diameter groove of the dual-diameter pulley, respectively; θ is the pulley rotation angle; and Δl denotes the total contraction of the series-connected SMA drive modules on one side.

According to Equations (1) and (2), under constrained geometric dimensions of the joint system, the differential joint achieves two critical advantages: ➀ increasing SMA actuator contraction (Δl) via the fixed pulley assembly, and ➁ enhancing motion amplification ratio by replacing the conventional pulley radius *R* with the equivalent radius $r_{a}-r_{b}$.

For example, when $r_{a}={2r}_{b}=R$, the equivalent radius is halved. If the SMA actuator length remains constant, the design of the pulley group increases the number of serially connected driving modules, causing the corresponding Δl to double. Consequently, the final joint stroke expands to four times the original range.

**Figure 3:**
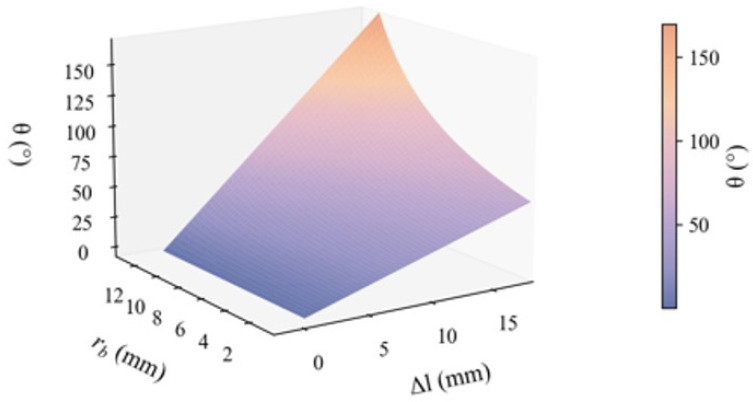
Maximum stroke variation diagram of the joint.

Building on Equation (2), by fixing the large-diameter groove radius $r_{a}=$ 18 mm, we quantitatively investigate the relationship between the joint travel angle θ, the total contraction Δl of SMA drive modules, and the small-diameter groove radius $r_{b}$. As shown in Figure 3, when $r_{b}$ approaches $r_{a}$, the maximum joint travel angle θ is drastically amplified because the denominator term $r_{a}-r_{b}$ approaches zero. Conversely, when $r_{b}=0$, the dual-diameter pulley reduces to a conventional single-diameter pulley configuration, and the motion amplification effect vanishes. To quantify this behavior, we define the travel amplification coefficient:

(3) $$k=\frac{r_{a}}{r_{a}-r_{b}}$$

This coefficient directly correlates the geometric parameters of the pulley with its kinematic performance, thereby establishing a theoretical foundation for standardized joint design and analysis.

**Figure 4:**
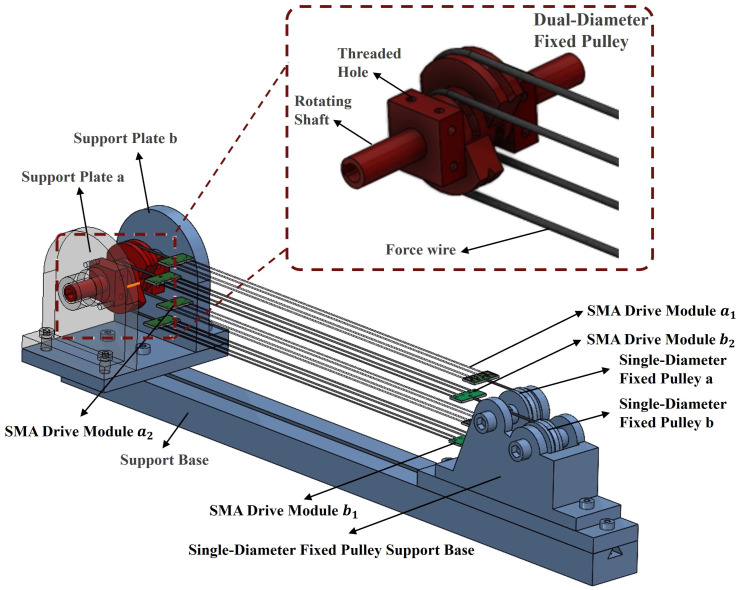
Schematic diagram of the overall joint structure.

Meanwhile, since excessively large unidirectional joint rotation angles would require more complex structural designs to avoid interference, we designed a dual-diameter pulley with a large groove radius of 18 mm and a small groove radius of 9 mm, achieving a motion amplification factor of 2. The radius parameter of the single-diameter fixed pulley is 27 mm. It increases the number of SMA drivers with multiple series connections on one side. Based on the integrated design combining multi-wire parallel SMA drivers, antagonistic actuator configuration, and differential dual-diameter fixed pulleys, we constructed the complete bio-inspired joint structure shown in Figure 4.

## 4. Implementation of Control Strategy and Bionic Joint Tracking Experiment

### 4.1. Adaptive Fuzzy Control Strategy

Due to the inherent nonlinearity and dynamic parameter variations of SMA, the system model constructed from the constitutive model of SMA cannot fully reflect the complete system knowledge, as it is merely a simplified representation of the actual controlled plant. Therefore, we adopt an adaptive fuzzy control strategy to bypass the challenges of system modeling while ensuring adaptability to system dynamics. Adaptive fuzzy control improves upon PID control by dynamically adjusting PID parameters through fuzzy inference to accommodate system variations. It comprises three steps: fuzzification, fuzzy inference, and defuzzification. We employ triangular membership functions for fuzzification, construct fuzzy rule tables based on experimental experience, and apply the centroid method for defuzzification. The updated PID parameters $K_{p}$, $K_{i}$ and $K_{d}$ are calculated as

(4) $$\left\{\begin{array}{c} K_{p}=\Delta K_{p}+K_{p_{0}}\\ K_{i}=\Delta K_{i}+K_{i_{0}}\\ K_{d}=\Delta K_{d}+K_{d_{0}} \end{array}\right.$$

where $K_{p_{0}}=0.3$, $K_{i_{0}}=0.001$, and $K_{d_{0}}=0.005$ are the initial PID parameters. We set the affiliation function to be a triangular affiliation function and use the center of gravity method for defuzzification. Real-time adjustments to the PID parameters based on $\Delta K_{p}$, $\Delta K_{i}$ and $\Delta K_{d}$, are implemented to enhance control performance.

### 4.2. Control System Architecture

**Figure 5:**
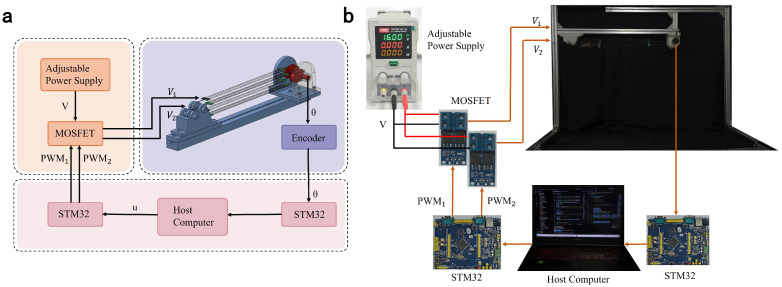
System architecture overview joint structure: (**a**) the rotation angle of the dual-diameter pulley is measured in real time by a high-precision absolute encoder(BRT38-5V5M), converted via ADC on an STM32F407ZGT6 microcontroller, and transmitted to a host PC via the RS232 protocol. The control algorithm, deployed on the host PC, generates PWM duty cycle commands sent to another STM32 module. This module outputs two PWM signals ($PWM_{1}$ and $PWM_{2}$) to MOSFETs, driving two independent voltage outputs ($V_{1}$ and $V_{2}$) for the independent thermal excitation and control of bilateral SMA driving modules. The adjustable power supply operates at a fixed voltage of 16 V. This framework not only validates control strategies for the current joint system but also provides an extensible foundation for future complex bio-inspired systems. (**b**) Physical configuration of the experimental setup.

The bio-inspired joint system is a complex integration of hardware and software. To coordinate subsystems and achieve precise control, a comprehensive framework is essential. For hardware architecture, the system adopts the distributed design shown in Figure 5a. The physical platform of the control system is shown in Figure 5b, with all components organized according to the diagrammatic connections.

### 4.3. Angle Tracking Experimental Results and Analysis

To validate the performance of the joint system, angle tracking experiments were conducted using step and sinusoidal reference input.

**Figure 6:**
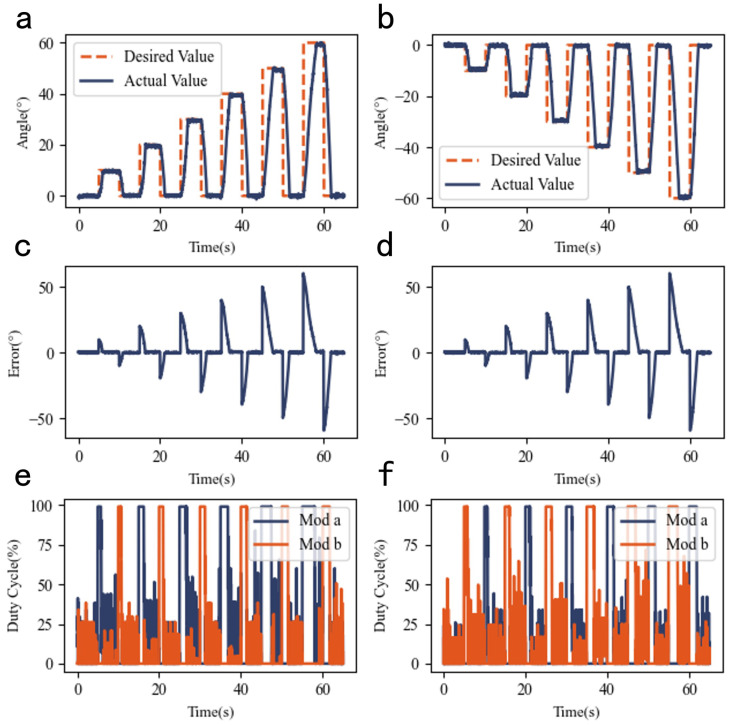
Step response characteristics of joint system: (**a**) Forward step response. (**b**) Reverse step response. (**c**) Forward step tracking error. (**d**) Reverse step tracking error. (**e**) Forward step PWM duty cycle. (**f**) Forward step PWM duty cycle.

For step reference inputs, two sets of bidirectional step signals were designed to verify the maximum designed stroke of joint (±60°) and bidirectional motion capability, as well as evaluating the dynamic coordination of the antagonistic actuator configuration. The results are shown in Figure 6.

The joint system achieved accurate convergence for step inputs of all magnitudes, demonstrating the feasibility and effectiveness of the control framework, including implementation of the control strategy, controller architecture, and hardware circuit calibration. For large-step inputs (e.g., 60°), the system converged rapidly, confirming the success of the differential dual-diameter joint design in achieving ±60° bidirectional motion and a total stroke of 120°.

To comprehensively assess the system’s ability to track periodic dynamic inputs, sinusoidal tracking experiments were performed to investigate the effects of amplitude and frequency on control performance. Two experimental groups were designed: ➀ Fixed frequency (0.05 Hz) with amplitude increasing from 10° to 60° in increments of 10°. ➁ Fixed amplitude (30°) with frequency increasing from 0.025 to 0.2 Hz.

**Figure 7:**
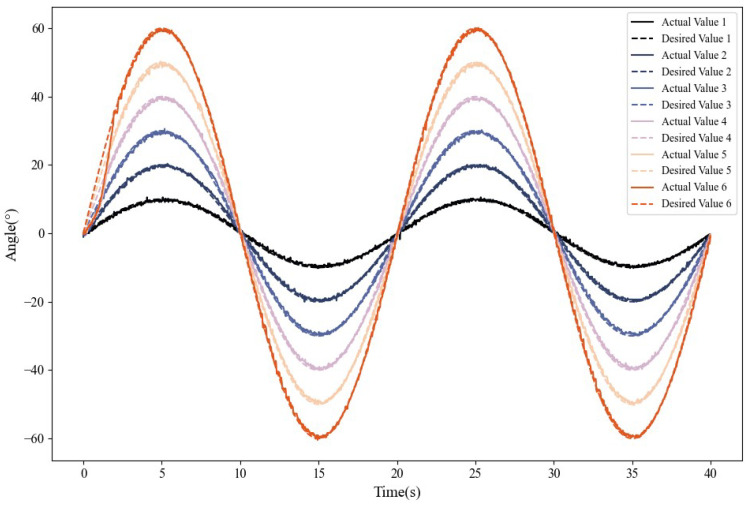
Sinusoidal tracking performance at specified frequencies.

Figure 7 shows the experimental results for varying amplitudes at a fixed frequency, demonstrating the accurate tracking of sinusoidal references across all the amplitudes.

**Figure A1:**
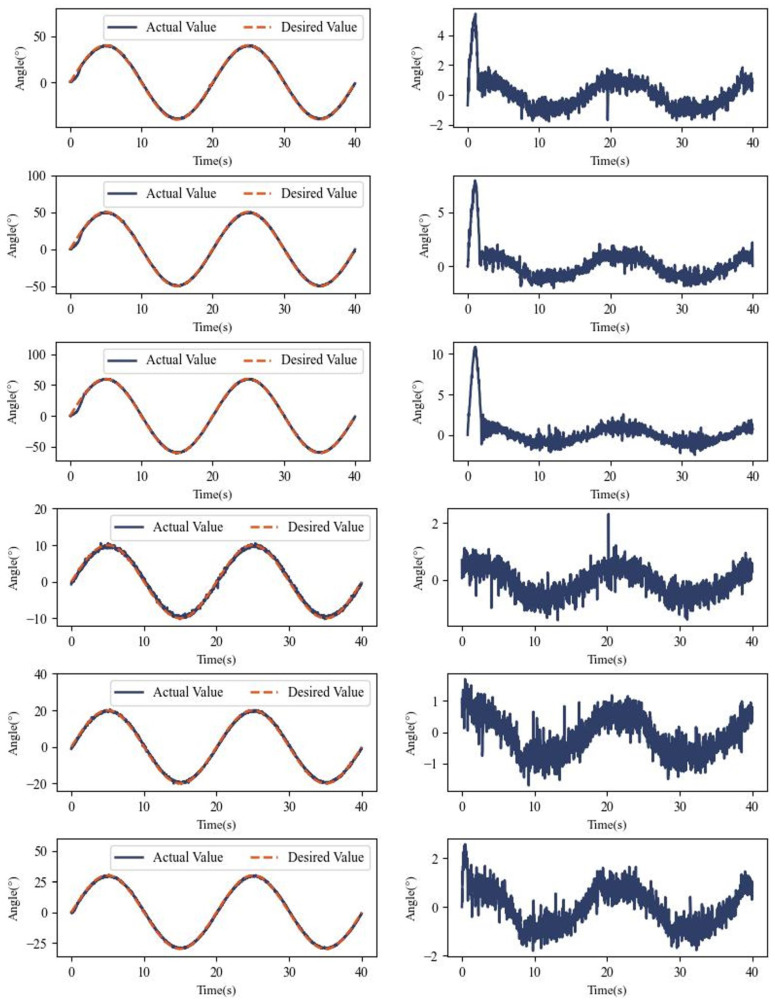
Sinusoidal tracking performance at specified frequencies.

Specifically, we show the tracking and error variation for each experiment in Figure A1 in Appendix A, which gives a better picture of the performance of the bionic joint.

Table 2 lists the root mean square errors (RMSEs) for different amplitudes. At 10° amplitude, the RMSE is as low as 0.48°, with errors gradually increasing at higher amplitudes while remaining within acceptable limits.

**Figure 8:**
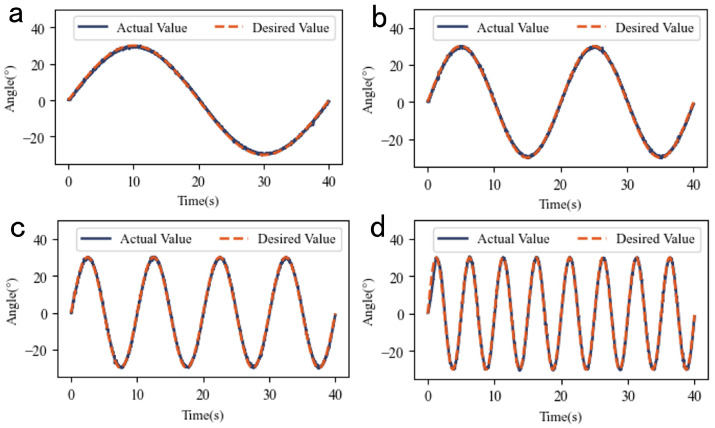
The experimental results of the sinusoidal reference input at different frequencies with fixed amplitude: (**a**) Actual vs. desired values at 0.025 Hz. (**b**) Actual vs. desired values at 0.05 Hz. (**c**) Actual vs. desired values at 0.1 Hz. (**d**) Actual vs. desired values at 0.2 Hz.

Figure 8 shows the experimental results for sinusoidal reference inputs with fixed amplitude and varying frequencies.

Table 3 lists the RMSE data for fixed-amplitude experiments, with a minimum value of 0.62°, demonstrating robust tracking performance.

Through step response and sinusoidal tracking experiments, the performance boundaries of the joint system were quantitatively evaluated. The experiments show that the system achieves overshoot-free position control within ±60°, with a maximum stroke of 120°. For mid-to-low frequency inputs (frequency < 0.2 Hz), the RMSE remains stable within 1°, meeting the precision requirements for bio-inspired joints. This validates the predefined performance metrics of the bio-inspired joint and provides a foundation for the subsequent development of the bio-inspired leg system.

### 4.4. Performance Evaluation of the Bio-Inspired Joint

Through experimental testing, the SMA-driven bio-inspired joint designed in this study demonstrates advantages in core performance metrics such as trajectory tracking accuracy and motion range. To objectively evaluate its technical advancement, this section introduces spatial utilization efficiency as a novel evaluation metric in addition to conventional indicators:

(5) $$\zeta =\frac{\theta_{max}}{L_{0}}$$

where $\theta_{max}$ is the maximum joint stroke, and $L_{0}$ is the longitudinal length of the SMA driving module. This metric quantifies the actuation capability per unit space, providing critical guidance for SMA-driven bio-inspired joints in robotic systems under spatial constraints. Table 4 compares the performance parameters of this system with existing studies.

Compared to similar works, our joint system achieves the following performance advantages.

Expanded Motion Range: The maximum rotation angle of 120° significantly surpasses most of other solutions listed in Table 4, validating the effectiveness of the differential pulley group design. While some of the works in the table also show travel beyond what the actuators designed in this paper have, such as the work of Park B. and A. Nespoli, these designs are not combined with control algorithms to obtain precise control effects that are difficult to apply to real robotic systems. In contrast, our core innovation of stroke amplification through the mechanism of a double-diameter pulley acting in concert with a pulley block still demonstrates effectiveness.

Superior Tracking Precision: In sinusoidal tracking experiments with amplitude of 30° and frequency of 0.025 Hz, the joint system achieves an RMSE of 0.62°, while the encoder resolution is 0.35°. This highlights excellent tracking accuracy, attributable to the antagonistic configuration enabling bidirectional active control and the dynamic tuning capability from adaptive fuzzy control.

Enhanced Spatial Utilization: Our joint system exhibits competitive spatial efficiency. By further increasing joint stroke ($\theta_{max}$) through adjustments to $r_{a}-r_{b}$, the spatial utilization efficiency can exceed the 75% reported in work of Zhu Y D et al.

Additionally, the modular and reconfigurable multi-wire parallel SMA driving module ensures scalability: parallel wire configurations accommodate high-load scenarios, while serial module extensions enable large-stroke applications.

For a realistic scenario, the measurement and estimation of the output force of the whole bionic joint or bionic leg is very important. For the SMA filaments we used, the output force is 34.93 N in the state of four parallel connections; meanwhile, due to the reconfigurability of the SMA driver module we used, the output force can be increased by increasing the number of parallel SMA filaments in order to achieve the adaptability to different working conditions. After that, we will add force sensors for the bionic joint to realize the accurate measurement and control of the output force to make the whole joint more integrated and complete.

In summary, the proposed joint system demonstrates the potential to achieve a large motion range with high precision, enabling the accurate replication of complex motions such as human-like gait. Its compact spatial integration addresses the critical need for deploying robotic actuators in confined spaces.

## 5. Bio-Inspired Leg System and Experiments

### 5.1. Bio-Inspired Leg System Construction

**Figure 9:**
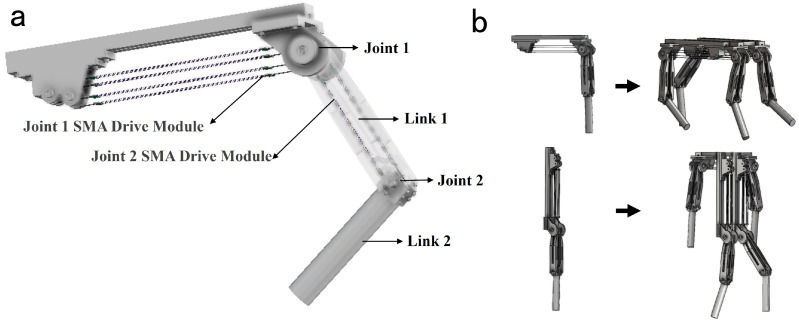
Bionic leg schematic diagram and two assembly methods: (**a**) Bionic leg structural. (**b**) Two assembly methods and their corresponding robotic configurations: quadruped and humanoid robots (schematic diagrams included).

This study adopts a rotating joint and rigid linkage architecture to construct the bio-inspired leg system. As shown in Figure 9a, the leg schematic includes two degrees of freedom: the hip joint module inherits the antagonistic differential joint from previous sections, while the knee joint employs a simplified single-diameter fixed pulley design with a no-load rotational stroke of 50°. Similarly to the antagonistic differential joint, the knee joint also utilizes an antagonistic actuator configuration and multi-wire parallel SMA driving modules as its power source.

Building on the modular control framework established earlier, the bio-inspired leg system was integrated with minimal hardware modifications and direct software reuse, validating the scalability and engineering practicality of original architecture. The control system and the actual hardware configuration of the leg are similar to those of the joint shown in Figure 5 but with two key extensions: ➀ A 10-bit absolute encoder (BRT38 series) is installed at Joint 2 to measure its rotation angle. ➁ Two additional MOSFET driver circuits are added to enable four-channel independent voltage output.

Figure 9b illustrates two distinct motion modes of the bio-inspired leg, determined by the assembly configuration of link1 on joint1. When link1 is perpendicular to the base platform, the system operates in quadruped mode; when link1 is parallel to the base platform, it switches to humanoid mode. It is important to note that this diagram primarily demonstrates the potential of the leg for integration into robots with higher degrees of freedom. The depicted quadruped and humanoid robots are not mature designs.

### 5.2. Bio-Inspired Leg System Construction

**Figure 10:**
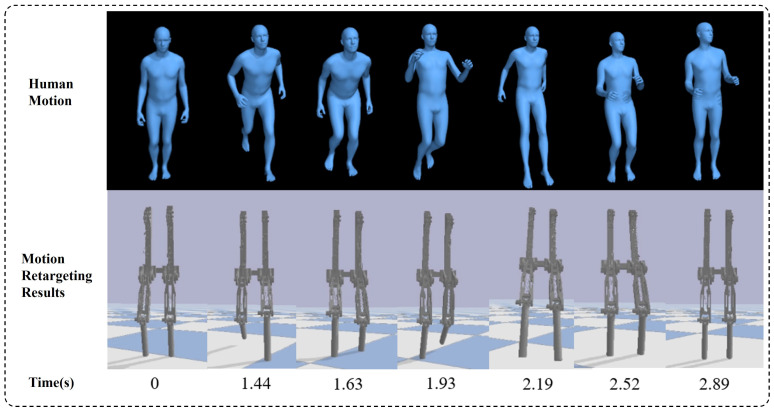
Motion retargeting results.

A two-step optimization motion retargeting method, inspired by H2O [28], is used to map human motions from the AMASS dataset [29] and other ways to the bio-inspired leg. To improve retargeting accuracy and avoid motion sequence jumps caused by local optima in numerical optimization, the second-step optimization equation is redesigned as follows:

(6) $$\begin{array}{c} \underset{q^{robot}}{min}\;\left(\sum_{i}\;w_{i}^{p}{∥p_{i}^{smpl}\left(\theta ,\beta^{\prime }\right)+P^{\prime }-p_{i}^{robot}\left(q^{robot}\right)∥}_{2}\right)\\ \;\;\;\;\;\;\;\;\;\;+\left(\sum_{j}\;w_{j}^{R}{∥R_{j}^{smpl}\left(\theta ,\beta^{\prime }\right)-R_{j}^{robot}\left(q^{robot}\right)∥}_{F}\right)\\ \;s.t.\;\;\;q^{robot}\in A \end{array}$$

where $w_{i}^{p}$ and $w_{j}^{R}$ are weighting coefficients, θ represents the human pose parameters per frame, $p_{i}^{smpl}$ and $p_{i}^{robot}$ denote the global positions of the key point *i*, $R_{j}^{smpl}$ and $R_{j}^{robot}$ are the rotation matrices of the link-fixed coordinate systems for key point *j*, and *A* is the joint space considering mechanical limits. The optimization combines position and rotation terms: position differences are measured by the $L_{2}$ norm, while rotation discrepancies use the Frobenius norm. Compared to H_2_O, our method incorporates rotation terms for key points to mitigate abrupt joint angle changes caused by position-only optimization. Figure 10 shows the motion retargeting results of a bipedal robot in simulation.

### 5.3. Gait Tracking Experiments and Analysis

**Figure 11:**
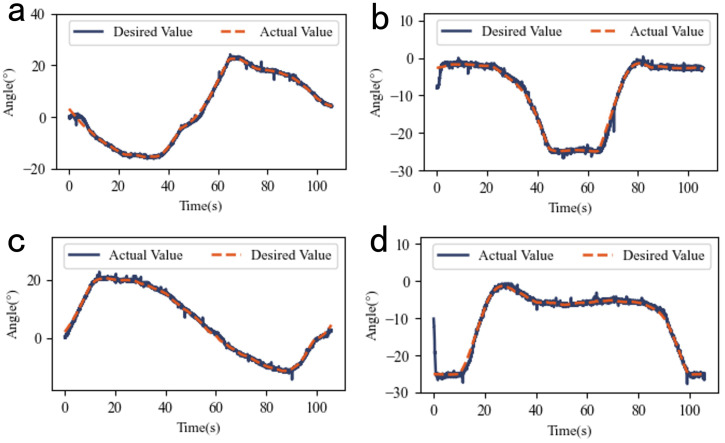
Human motion tracking experiment. (**a**) Tracking of the hip joint in the left leg motion retargeting results by bionic leg joint 1. (**b**) Tracking of the knee joint in the left leg motion retargeting results by bionic leg joint 2. (**c**) tracking of the hip joint in the right leg motion retargeting result by bionic leg joint 1. (**d**) tracking of the knee joint in the left leg motion retargeting result by bionic leg joint 2. It is worth noting that the experimental data shown in figures (**a**) and (**b**) were obtained in a single experiment, as were the experimental data shown in figures (**c**) and (**d**).

Since the designed leg is a single-leg system without chirality, two experiments were conducted to track the redirected motions of the left and right human legs. The results are shown in Figure 11.

For left leg tracking, RMSE values were 0.68° (left hip), 0.77° (left knee), 0.57° (right hip), and 0.97° (right knee). The small RMSE values confirm the ability of the leg to replicate biological motions and validate the feasibility of the SMA-driven joint system.

We recognize that the current design is imperfect. For example, when tracking normal gait data (with a period of about two seconds), the experimental results are not satisfactory due to the immature design of the bionic leg structure. This problem can be solved in two ways, on the one hand, with better bionic leg design, especially the optimization of linkage structure, and on the other hand, by increasing the number of parallel filaments in the drive module. Nevertheless, our initial intention has been achieved, i.e., through structural innovation and algorithm deployment, we have initially explored the application of the SMA drive scheme in footed robotic systems, and in future work, we will launch a more in-depth study and verify the advantages of the SMA drive scheme through more detailed experiments.

**Figure 12:**
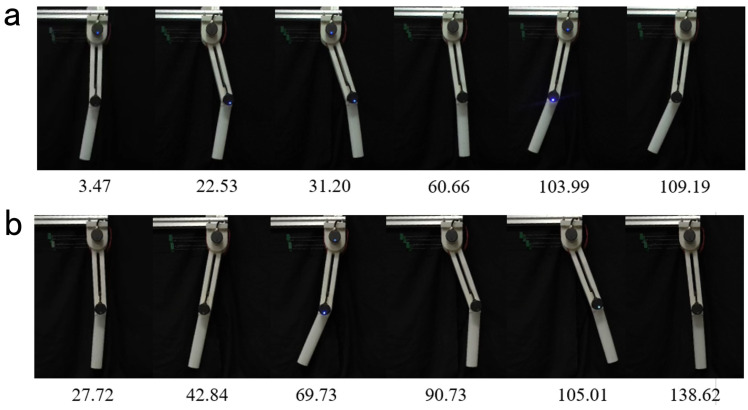
Lower limb motion tracking experiment. The numbers below the experimental image show the moment of the current frame in seconds. (**a**) Physical diagram of the left leg redirection result tracking experiment. (**b**) Physical diagram of the right leg redirection result tracking experiment.

Figure 12 demonstrates the physical leg platform executing a full gait cycle: The left leg swings forward (swing phase) and backward (stance phase), while the right leg follows the reverse sequence. The smooth tracking of the reference input, such as human-like motion, further verifies the potential of the the bionic leg and the correctness of the motion retargeting method.

## 6. Conclusions

This study systematically investigates the SMA-driven and bio-inspired joint and leg, covering actuator design, joint structural innovation, control strategy deployment, and system integration, establishing a complete technical chain from fundamental actuation units to application validation. Experimental results demonstrate that the antagonistic actuator configuration and differential dual-diameter fixed pulley design not only enable bidirectional active control but also surpass traditional SMA-driven joints in motion range. The bio-inspired leg prototype, integrating these joints and control systems, successfully replicates human gait dynamics, validating the performance of the joint and offering a potential actuator solution for robotics.

In the future, building upon the bio-inspired joint and leg systems explored in this work, several directions warrant further investigation. For joint structures, more mature designs are needed to ensure stable system operation. In control algorithms, learning-based methods should be explored to achieve superior performance. At the foundational level, developing more robust joints, and at the application level, constructing bio-inspired robots with higher degrees of freedom, will advance the practical deployment of this technology in real-world scenarios.

## Figures and Tables

**Table 1:** Dimensional parameters of multi-wire parallel SMA drive module.

Parameter	Value (Unit)
Dimensions of Multi-Wire Parallel SMA Drive Module	320 mm × 20 mm × 1.6 mm
Dimensions of PCB1	20 mm × 10 mm × 1.6 mm
Dimensions of PCB2	20 mm × 10 mm × 1.6 mm
SMA Wire Length	300 mm
Number of SMA Wires	4

**Table 2:** RMSE results at fixed-frequency sinusoidal input.

Amplitude (°)	RMSE (°)
10°	0.48
20°	0.62
30°	0.78
40°	1.04
50°	1.40
60°	1.80

**Table 3:** RMSE data at fixed-amplitude sinusoidal reference input.

Frequency (Hz)	RMSE (°)
0.025	0.62
0.05	0.73
0.1	0.93
0.2	1.66

**Table 4:** Comparison of the performance of several SMA rotary actuators.

Paper	SMA Drive Module Length (mm)	Maximum Rotation Angle (°)	RMSE (°)	Spatial Utilization Efficiency (%)
JIA Z K [26]	380	62	-	16.32
Zhu Y D [11]	80	60	0.89	75
BRITZ R [27]	196	30	-	15.31
GUO Z [14]	370	30	0.5	8.11
Park B [3]	-	90	-	-
A. Nespoli [7]	20	160	-	800
ours	300	120	0.62	40

## Data Availability

Data are contained within this article.

## Appendix A

Figure A1 illustrates the experimental results when the amplitude is varied with a fixed reference input frequency, which can be viewed as an independent subplot of Figure 7.
